# An Extended Energy-Biased Aggregation-Volume-Bias Monte Carlo (EB-AVBMC) Method for Nucleation Simulation of a Reactive Water Potential

**DOI:** 10.1021/acs.jctc.5c00722

**Published:** 2025-07-04

**Authors:** Anthony Val Canillas Camposano, Even Marius Nordhagen, Anders Malthe-So̷renssen, Henrik Andersen Sveinsson

**Affiliations:** † The Njord Centre, Department of Physics, 6305 University of Oslo, 0316 Oslo, Norway; ‡ 114311 Norwegian Meteorological Institute, 0313 Oslo, Norway

## Abstract

The aggregation-volume-bias Monte Carlo (AVBMC) algorithm has been widely used with empirical water models like TIP3P, SPC/E, TIP4P, and TIP4*P*/2005 to study nucleation and vapor–liquid properties, but its application to reactive water models remains underexplored. Here, we present an extension of the energy-bias aggregation-volume-bias Monte Carlo (EB-AVBMC) method for calculating nucleation free energies and liquid–vapor properties, such as gas density and surface tension, using a three-body reactive force field based on the Vashishta potential functional form [*Phys. Rev. B* **1990**, *41*, 12197–12209]. Key modifications include revised acceptance rules that consider the intramolecular energy of the inserted/deleted molecule to prevent high acceptance probabilities that could bias the sampling and constraints to avoid the deletion of dissociated water molecules. These adjustments ensure valid bond topology modifications. We demonstrate the method’s applicability by studying water nucleation at 298.15 K, with varying cluster sizes, and showing a free energy consistent with studies from rigid water models. This approach is generalizable to other reactive water force fields, offering a valuable tool for simulating reactive liquid–vapor properties.

## Graphical abstract

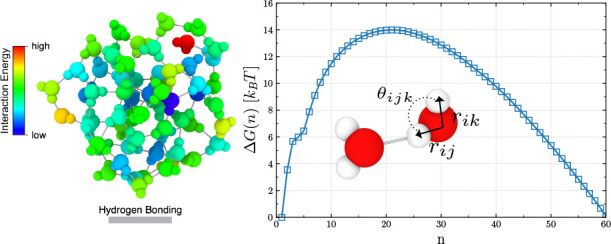

## Introduction

Nucleation is a fundamental process involved in various physical and chemical phenomena, impacting a wide range of fields such as atmospheric science,^1^ material science,^2,3^ and nanotechnology.^4,5^ The study of nucleation serves as a powerful tool for understanding and controlling phase transitions in a wide array of systems. Nucleation is the initial process in a phase transition where a new, thermodynamically stable phase begins to form within a metastable mother phase. A common example is vapor–liquid nucleation, where liquid droplets form in supersaturated vapor. Nucleation events, involving the formation of a new phase from a metastable one, are inherently rare and typically require long time scales to observe, making their study challenging.

Simulation techniques like molecular dynamics and Monte Carlo simulations allow for studying nucleation at the molecular and atomic level.^6–17^ However, traditional molecular dynamics simulations often struggle to capture nucleation events in some systems due to the substantial free energy barriers separating the metastable and new phases.^18^ For instance, sampling strongly associating fluids presents a significant challenge.^11,16,17^ The use of traditional methods like the standard Metropolis Monte Carlo also struggles with these systems because they rely on a symmetric Markov matrix, making it difficult to explore rare bonded states in a vast configuration of nonbonded states. Breaking bonds in such systems requires a large amount of energy, further complicating the escape from these low-energy bonded states. To address these limitations, biased algorithms that favor bond formation have been developed.^6,8,12,14,19^ These biases must be carefully corrected in the acceptance rule to ensure accurate results. Different biased algorithms have their own advantages and limitations. Association-biased Monte Carlo (ABMC)^20,21^ is complex and computationally expensive, requiring predefined bonding regions. Bond-bias Monte Carlo (BBMC)^22^ is simpler but limited to specific bonding characteristics. Monomer-addition-subtraction (MASA)^6,23^ is more general, handling chain-forming molecules, but is less efficient and limited in the types of bonding it can handle.

The aggregation-volume-bias Monte Carlo (AVBMC) algorithm^11,24^ seeks to alleviate some of these shortcomings. It enhances the efficiency of sampling particle exchanges between free monomers and clusters and between two clusters. By focusing on a small region around a given molecule, AVBMC increases the likelihood of generating bonded configurations, improving both the bond formation and destruction rates. In nucleation simulations, AVBMC eliminates the need for a finite-volume simulation box and has been implemented for various ensembles, such as the canonical,^11,24^ isobaric–isothermal, and grand canonical ensembles.^12^ Kusaka et al.^10^ were the first to use the grand canonical ensemble for nucleation simulations where the system contains a physical cluster and a fictitious ideal gas; it is particularly efficient for associating fluids like water, where molecules form clusters through strong interactions such as hydrogen bonding. This method allows for sampling cluster size distributions via particle insertions or removals, accounting for both energetic and entropic factors. It avoids the arbitrary choice of cell size and is computationally efficient, especially in systems prone to trapping in stable bonded states with high Boltzmann weights. A good simulation method must efficiently sample both the low-energy configurations associated with strong bonding and the larger phase space of nonbonded configurations. To enhance the exploration of phase space, the AVBMC algorithm introduces a novel trial move called the intrabox swap move, analogous to the particle swap moves in the grand canonical and Gibbs ensembles. This algorithm includes two corresponding moves: insertion (swap-in) and deletion (swap-out), allowing for particle exchange between the system and its surroundings.

Another critical problem is overcoming the large free energy barrier. To overcome this, enhanced sampling techniques such as umbrella sampling^25^ are frequently employed. In several nucleation simulation studies,^12,14,26^ the AVBMC is coupled with self-adaptive umbrella sampling to solve the critical problem of sampling high free-energy clusters. The AVBMC algorithm accelerates particle transfer between clusters and the mother phase, while the umbrella sampling introduces biasing potentials that significantly enhance the likelihood of visiting these critical clusters, speeding up the sampling process and improving the accuracy of nucleation simulations. Umbrella sampling is straightforward to implement in Monte Carlo simulations, requiring only modifications to the acceptance rules; however, it can still encounter bottlenecks, particularly in dense or confined systems within grand canonical ensembles.

A significant drawback of the original AVBMC algorithm is that it typically produces low acceptance rates, often below 1%, for instance, in simulations of TIP4P water.^27^ Consequently, a significant amount of computational time is spent on rejected moves, limiting efficient exploration of the relevant phase space. While the introduction of an energy biasing technique improved this to 4–6%, the acceptance rate remains low, especially in systems with a large number of particles. To further enhance efficiency, Loeffler et al.^28^ proposed an improved AVBMC algorithm that biases both the insertion and removal of particles to favor locations conducive to cluster growth, such as the surfaces of existing clusters. This refined approach, known as energy-biased aggregation-volume-bias Monte Carlo (EB-AVBMC), significantly improves the sampling efficiency from 4 to 6% to 20–35%. The EB-AVBMC technique has proven particularly valuable for studying the nucleation of strongly associating molecules, such as water, which exhibit complex hydrogen bonding networks.

In addition, Loeffler et al.^29^ demonstrated that EB-AVBMC is effective in accurately estimating vapor–liquid properties for water, notably enhancing the performance of the TIP3P model. Their results suggest that EB-AVBMC can serve as an indirect tool to parametrize water models, improving agreement with experimental data for properties such as boiling point, critical temperature, and surface tension. Numerous studies have employed AVBMC with classical water models like TIP3P,^29^ SPC/E,^11^ and TIP4P^27^ to investigate nucleation and vapor–liquid properties, but its application to reactive water models remains relatively unexplored. Kumar et al.^30^ applied EB-AVBMC to multistate empirical valence bond (MSEVB) models for ion-induced nucleation but did not address homogeneous nucleation in dissociative water models. This gap motivates exploring EB-AVBMC for reactive systems to better capture liquid–vapor behavior in dissociative water models.

In this work, we extend and adapt the EB-AVBMC method for use with a dissociative, all-atom water model based on the Vashishta potential functional form.^31^ The modifications include tailored acceptance criteria and constraints to handle the changes in bond topology associated with dissociative behavior. We achieve acceptance ratios of 4–6% for AVBMC moves and 35% for single-atom translation moves, ensuring efficient sampling of configurational space. We demonstrate the feasibility and performance of this enhanced method by simulating water nucleation at 298.15 K. Our results show that this approach can reliably capture nucleation events, enabling its potential application to other reactive water models.

## Interatomic Potential Model

Running EB-AVBMC calculations on atomic systems requires a potential energy to be computed for each trial move. This energy is derived from interatomic interactions within a cluster of 3*N* atoms (*N* is the number of water molecules) and is crucial for accurately capturing the microscopic details of nucleation. Here we model these interactions using the Vashishta potential,^32,33^ a well-established form for describing multibody interatomic forces, which is suitable for modeling reactive water. The total energy of the system is expressed as

$$U=\overset{N}{\underset{i}{\sum }}U_{i}$$

where the interaction energy of atom *i* is given by,

$$U_{i}=\sum_{j>i,i\neq j}^{N}U_{ij}^{\left(2,S\right)}(r_{ij})+\sum_{j\neq i}^{N}\sum_{k>j,k\neq i}^{N}U_{ijk}^{\left(3\right)}(r_{ij},\;r_{ik},\;\theta_{jik})$$

The two body terms are shifted for *r* < *r*_c_ and truncated at the cutoff *r*_c_, making energy and forces at the cutoff continuous. The expression of the truncation and shift is given by,

$$U_{ij}^{\left(2,S\right)}(r)=\{\begin{array}{ll} U_{ij}^{\left(2\right)}(r)-s(r)-t & \left(r<r_{c}\right)\\ 0 & \left(r\geq r_{c}\right) \end{array}$$ 1

where

$$t=U_{ij}^{\left(2\right)}(r_{c})$$ 2

$$s(r)=(r-r_{c}){\left(\frac{dU_{ij}^{\left(2\right)}(r)}{dr}\right)}_{r=r_{c}}$$ 3

The two-body terms consist of steric repulsion, Coulomb interaction, charge-dipole interaction, and an additional term for the van der Waals interaction. The two-body terms are expressed as

$$U_{ij}^{\left(2\right)}(r_{ij})=\frac{H_{ij}}{r_{ij}^{\eta_{ij}}}+\frac{Z_{i}Z_{j}}{r_{ij}}e^{-r_{ij}/r_{1s}}-\frac{D_{ij}}{r_{ij}^{4}}e^{-r_{ij}/r_{4s}}-\frac{W_{ij}}{r_{ij}^{6}}$$ 4

for *r*_*ij*_ ≤ *r*_c_. These terms are summed over all neighbors of atom *i* within a cutoff range *r*_c_. The parameter *r*_1*s*_ is the screening length of the Coulomb interaction, and *r*_4*s*_ is the screening length of the charge-dipole interactions. The free parameters *H*_*ij*_, *Z*_*i*_, *D*_*ij,*_ and *W*_*ij*_ are the steric repulsion strength, the effective charge, the charge-dipole interaction strength, and the van der Waals attraction strength, respectively. The η_*ij*_ is the exponent of the steric repulsion.

The three-body term consists of factors involving bond stretching *f*(*r*_*ij*_, *r*_*ik*_) and bond bending *p*(θ_*jik*_, θ_*jik*_^0^),

$$U_{ijk}^{\left(3\right)}(r_{ij},\;r_{ik},\;\theta_{jik})=B_{ijk}f(r_{ij},\;r_{ik})p(\theta_{jik},\;\theta_{jik}^{0})$$ 5

where the parameter *B*_*jik*_ modulates the strength of the three-body potential. The stretching function ensures that the three-body force decreases with bond distance and vanishes at *r_0_*,

$$f(r_{ij},\;r_{ik})=\exp\left(\frac{\xi }{r_{ij}-r_{0}}+\frac{\xi }{r_{ik}-r_{0}}\right)$$ 6

where *r*_*ij*_, *r*_*ik*_ ≤ *r*_0_; otherwise, *f*(*r*_*ij*_, *r*_*ik*_) = 0, and the bending function is a harmonic function of the bending angle, given by

$$p(\theta_{jik},\;\theta_{jik}^{0})=(\cos\;\theta_{jik}-\cos\;\theta_{jik}^{0}{)}^{2}$$ 7

The three-body form applies to all H–O–H triples, for which the distance between the oxygen atom and each of the hydrogen atoms is less than *r*_0_. A screened cosine-squared function is used to model the angular dependence of the three-body interaction. The angles θ_*jik*_^0^ and θ_*jik*_ are the minimum angle and the H–O–H angle, respectively. Beyond the pairwise cutoff distance *r*_0_, the three-body term is zero due to the decaying exponential term. All parameters for the reactive water potential are provided in a Supplementary File.

## Reactive Water in EB-AVBMC

Our implementation of the AVBMC algorithm is largely based on the work of Loeffler et al.,^28^ with modifications to accommodate the dissociative nature of the Vashishta water potential. We added a bond topology update and a water monomer deletion constraint specifically to accommodate our all-atom dissociative water model in the AVBMC algorithm. In addition, we introduce new acceptance rules to take into account the intramolecular energies of a water molecule.

The general setup of the simulation is a grand canonical ensemble for computational efficiency.^10^ A separate cluster region and a gas phase system (fictitious ideal gas) make this simulation independent of the volume. The gas phase and cluster system are said to be “thermodynamically coupled,” where the gas phase system’s chemical potential μ is given in terms of gas number density ρ_v_. A Stillinger cluster criterion^34^ defines a cluster as a group of molecules in which every molecule has at least one neighbor in the group within a certain distance. In our simulations, we used a gas number density ρ_v_ = 6 × 10^–7^ Å^–3^ and a Stillinger criterion *r*_cl_ = 4 Å. To overcome the free energy barriers associated with cluster formation and efficiently sample the cluster size distribution, we employed the Umbrella Sampling method.^35^ In this approach, a biasing potential, which is a function of the chosen reaction coordinate, is added to the system’s Hamiltonian to enhance sampling in specific regions of phase space. The biasing potential is typically constructed as a series of overlapping windows, each centered on a specific value of the reaction coordinate. Within each window, the system is simulated, and the biased probability distribution is recorded. These individual, biased distributions are then combined using the weighted histogram analysis method (WHAM)^36^ to reconstruct the unbiased free energy profile along the reaction coordinate. For our simulations, the cluster size was used as the reaction coordinate. The WHAM analysis was performed with a convergence tolerance of 1 × 10^–7^, a maximum of 30,000 iterations, and using segments of 100,000 data points from each umbrella window. The biasing potential was iteratively refined,^12^ to make the sampling within each window more uniform.

### EB-AVBMC Moves

The Monte Carlo moves used, shown in Figure 1b–d, consist of water molecule swap moves and single atom translations, which we choose to perform at a ratio of 1:10. Insertion and deletion moves, which we perform with equal frequency, were carried out using 50 Rosenbluth trials. In the water molecule swap move, a bonded region is considered as a sphere with a 4 Å radius centered on a randomly selected oxygen atom from the cluster phase. This radius corresponds to the Stillinger cluster criterion, ensuring that each accepted swap move inherently satisfies this condition.^11,28^ For insertion, the water monomer is grown by first creating a hydrogen and an oxygen with a generated bond length *r*_1_ sampled using a Gaussian distribution fitted to the bond-length distribution from a molecular dynamics simulation of the gas phase of water at 298.15 K. Then the second hydrogen is generated by rotating a copy of the first hydrogen using an angle θ sampled from a Gaussian distribution fitted to the angle distribution of the gas phase of water at the same temperature. Its bond length is then adjusted to the sampled bond length *r*_2_ from the same Gaussian distribution used to generate *r*_1_. The complete water molecule is inserted at a random distance within the bonded region of the target oxygen atom. To enhance insertion efficiency, we employ Configurational-Bias Monte Carlo (CBMC),^37^ which generates *K* trial configurations, increasing the probability of finding an energetically favorable (lower energy) placement. Among these *K* generated trial configurations, one of them is randomly selected, and the probability of selecting each of them is given by their Rosenbluth factor

$$P_{k}=\frac{e^{-\beta U_{k}}}{W}$$ 8

where *P*_*k*_ is the probability of selecting configuration *k*, *U*_*k*_ is the total interaction energy of the trial configuration *k*, β ≡ 1/*k*_B_ *T* is the reciprocal temperature, and the normalization factor *W* is the total Rosenbluth weight,^38^

$$W=\sum_{k=1}^{K}e^{-\beta U_{k}}$$ 9

This way of sampling enhances the chance of an acceptable trial configuration by preferentially selecting configurations with lower energies. When a trial configuration *m* is selected, the Rosenbluth factor *P*_trial_^*m*^ is used to compute an acceptance criterion that compensates for the selection bias. The probability of selection *P*_*k*_ is scaled by the number of trials (*K*), each configuration’s probability is fairly weighted across the entire sampling process during the reverse move of inserting in the ideal gas phase reservoir; thus, we have,

$$P_{\mathrm{trial}}^{m}=\frac{e^{-\beta U_{m}}K}{W}$$ 10

**Figure 1:**
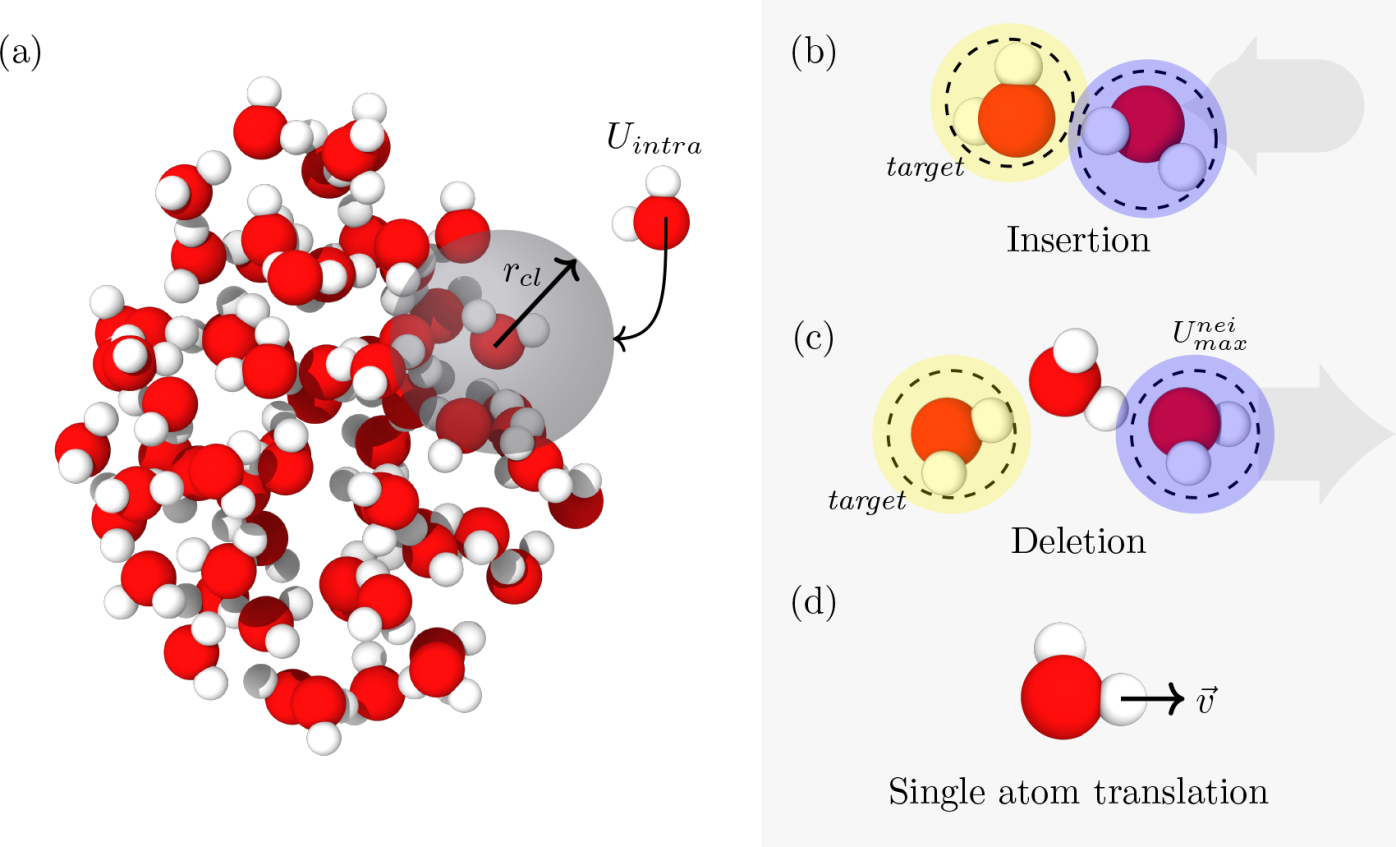
Snapshot of a water cluster and the Monte Carlo (MC) moves used in the algorithm. (a) State of a cluster after an MC move is characterized by its change in energy Δ*U* and the intramolecular energy *U*_intra_ of the inserted/deleted water molecule within a spherical region defined by the Stillinger criterion distance *r*_cl_. Panels on the right show the MC moves used. The EB-AVBMC insertion and deletion moves follow the algorithm of Loeffler et al.,^28^ but with modifications to take into account the dissociative nature of the Vashishta water potential. (b) Standard insertion move is a selection of a target molecule (yellow shade) and then inserting randomly within the spherical region of the target molecule another water molecule (blue shade). (c) In a deletion move, a target (yellow shade) is selected, and then one of its neighbors with the highest energy *U*_max_^nei^ is selected for deletion (blue shade). A constraint is introduced to reject deletion moves on a structural unit of water if one of its hydrogen is beyond 2 Å from its assigned oxygen. (d) Atom for translation is selected by first selecting a water molecule of the cluster and then selecting an atom in that selected water molecule.

From systematic studies of the TIP4P water model, Loeffler et al.^28^ found that accepted insertion moves are biased toward the surface of the water cluster. Therefore, the insertion acceptance ratio can be increased by biasing the insertion toward the surface. This can be achieved through the following selection probability for the energy of the target molecule:

$$P_{\mathrm{target}}^{I}=\frac{e^{\alpha U_{i}}}{\sum_{j}e^{\alpha U_{j}}}$$ 11

where *U*_*i*_ represents the sum of all energetic interactions of a target molecule *i* with every other molecule in the system, and α is a tuning parameter provided by the user to modulate the strength of the insertion target biasing. In our case, we use a value of 0.1β, as this was shown by Loeffler et al.^28^ to strike a balance between significantly improving insertion acceptance rates by targeting higher-energy molecules on the cluster surface without overly restricting the sampling to only a few specific sites. The probability of inserting this newly generated water molecule in a randomly selected point in the bonded region of the target molecule is

$$P_{\mathrm{select}}^{I}=1/V_{\mathrm{in}}$$ 12

where *V*_in_ = 4π*r*_cl_^3^/3.

Water monomer deletion follows the standard procedure of the AVBMC algorithm, where a molecule within a bonded region of volume *V*_in_ is selected first with probability *P*_target_^D^, then one of its neighbors is selected as the trial monomer for deletion with probability *P*_select_^D^. A constraint was introduced in this deletion method to account for the dissociative nature of the water molecules. The key condition for deletion is that the two closest hydrogen atoms in the system must be within a bond length of 2 Å from the oxygen atom being deleted. As shown in Figure 1c, during a deletion move, one water molecule is designated as the target (highlighted in yellow), and then another water molecule is randomly selected for deletion (highlighted in blue). The target molecule is selected with a probability of

$$P_{\mathrm{target}}^{D}=\frac{e^{\beta U_{i}}}{\underset{j}{\sum }e^{\beta U_{j}}}$$ 13

In the standard AVBMC algorithm, the molecule selected for deletion is not the initial target but one of its neighboring molecules. This ensures that molecules with higher energies are more likely to be chosen as deletion targets. The selection is biased by the highest energy of the *N*_nei_ neighboring molecules, *U*_max_^nei^, with the probability of deletion given by

$$P_{\mathrm{select}}^{D}=\frac{e^{\beta U_{\max}^{\mathrm{nei}}}}{\overset{N_{\mathrm{nei}}}{\underset{j}{\sum }}e^{\beta U_{j}}}$$ 14

This formulation biases the selection toward molecules with higher energies.

In rigid water molecules, as in the work of Loeffler et al.^28^ and Chen and Siepmann,^11^ the intramolecular energy of the water monomer is not used when computing energy differences, since all monomers are equal. In the Vashishta force field, since it is an all-atom model, we need to properly account for the intramolecular energy. In our all-atom simulations, the intramolecular energy *U*_intra_ of the target molecule is therefore subtracted during addition and added during deletion when the energy change from the old state energy *U*_old_ to the new state energy *U*_new_. This accounts for the binding energy of the monomer in the reservoir. A similar approach, taking into account the intramolecular energies, was employed in the acceptance probability of the reactive grand-canonical Monte Carlo simulations by Heijmans et al.^39^ The EB-AVBMC algorithm’s new acceptance rules in the grand canonical ensemble that we use are

$$P_{\mathrm{insert}}^{\mathrm{Acc}}=\frac{P_{\mathrm{remove}}}{P_{\mathrm{insert}}\cdot P_{\mathrm{trial}}^{m}}\cdot e^{-\beta (\Delta U-U_{\mathrm{intra}}-\mu +U_{\mathrm{bias}}})$$ 15

and

$$P_{\mathrm{remove}}^{\mathrm{Acc}}=\frac{P_{\mathrm{insert}}\cdot P_{\mathrm{trial}}^{1}}{P_{\mathrm{remove}}}\cdot e^{-\beta (\Delta U+U_{\mathrm{intra}}+\mu +U_{\mathrm{bias}})}$$ 16

where Δ*U* = *U*_new_ – *U*_old_, μ is the chemical potential of the ideal gas phase reservoir, *U*_bias_ is the biasing potential from the Umbrella Sampling method using an iterative procedure,^12^ and *U*_intra_ is the intramolecular energy of the water molecule that is to be inserted or deleted. The probability of *P*_trial_^1^ uses the Rosenbluth factor of the selected water molecule for deletion of the cluster. The expression for the *P*_insert_ and *P*_remove_ is given by,

$$P_{\mathrm{insert}}=P_{\mathrm{target}}^{I}\times P_{\mathrm{select}}^{I}$$ 17

$$P_{\mathrm{remove}}=P_{\mathrm{target}}^{D}\times P_{\mathrm{select}}^{D}$$ 18

The probability *P*_insert_ and *P*_remove_ still follows the original formulation of Loeffler et al.^28^ To enhance the sampling of rare events like cluster formation and destruction, the biasing potential was iteratively sampled using a self-adapting procedure called AVUS-HR,^11,12,14,40,41^ which combines histogram reweighting with the AVBMC and a self-adaptive umbrella sampling. A similar approach was used in this study with our extended EB-AVBMC algorithm.

We used standard translational moves for each atom. The maximum atom translation was set at 0.05 Å. The target atom is selected by first selecting a molecule randomly in the system, followed by the random selection of an atom from the selected molecule. The acceptance rule for the translation is

$$P_{\mathrm{translate}}^{\mathrm{Acc}}=e^{-\Delta U/k_{B}T}$$ 19

Only the change in energy Δ*U* after the translation is used in the Boltzmann expression to determine if the move will be rejected or accepted. Crucially, accepted translations often require topology updates, because the dissociative model allows hydrogen atoms to dynamically detach from one oxygen and associate with another. An automated algorithm (see Supporting Algorithm 1) handles these updates by iteratively reassigning hydrogens to the closest oxygens. This bookkeeping is important for ensuring consistency during deletion moves, where the identity of the bonded atoms must be unambiguous. It ensures deterministic assignments of which hydrogen atoms are assigned to which oxygen atoms at any given moment in the simulation. Figure 2a–c illustrates how translations trigger topology changes, such as hydrogen migrating between two water molecules or forming transient ionic species.

**Figure 2:**
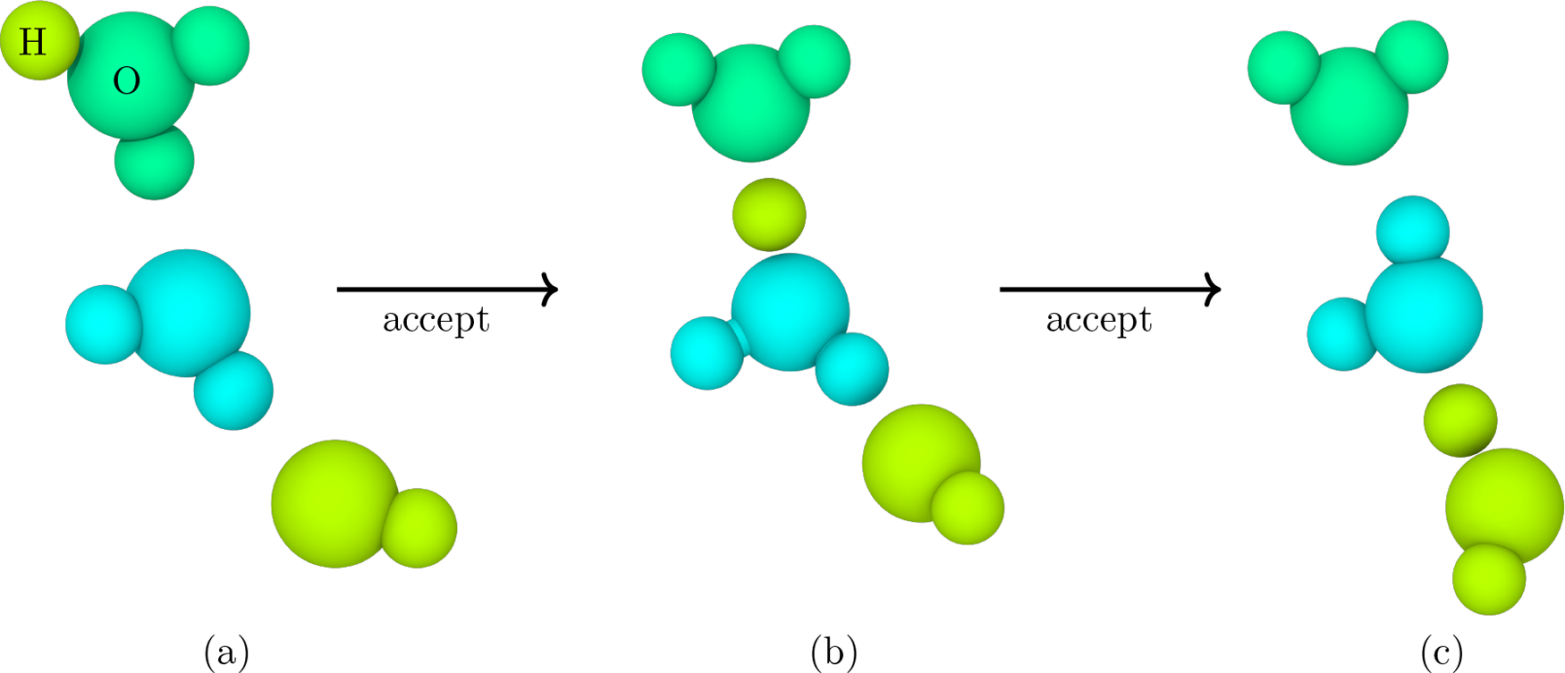
Dissociation of water using the Vashishta force field depicts the Grotthuss mechanism. Each atom is color-coded to show its associated molecule identifier. (a) While an oxygen atom can be surrounded by several hydrogen atoms, only two are assigned to that oxygen to form a single water molecule, with any additional hydrogens assigned to another oxygen atom. Following the acceptance of a translation move, the bond topology is updated for the subsequent cycle, as illustrated in (b) and (c). Each accepted translation leads to an update in the water system’s bond topology. This bookkeeping is useful in the AVBMC deletion moves in identifying the target water molecule.

## Results and Discussion

We evaluated the performance of our all-atom EB-AVBMC algorithm by running 128 parallel simulations on a water system at *T* = 298.15 K, using parameters of the Vashishta potential that we optimized in a previous study.^42^ Simulations were run for each of three maximum cluster sizes (30, 50, and 70), using 1.5 × 10^7^ Monte Carlo steps with 100 trial moves per step and a 1:10 ratio of AVBMC moves to translations. From these simulations, we obtained free energy changes associated with cluster formation.

The free energy of cluster formation, Δ*G*(*n*), and its incremental change, δΔ*G*(*n*), are central to understanding the nucleation behavior. Figure 3a,b shows the nucleation free energy profile of the simulation and the change of free energy due to the addition of a water molecule using the Vashishta potential for water. Figure 3a shows a free energy profile that is qualitatively consistent with results using a rigid water model.^10,27,28,43^ Each curve exhibits a characteristic free-energy barrier whose peak defines the critical cluster size. In line with classical nucleation theory (CNT), Δ*G*(*n*) increases monotonically at small *n* due to the growing interfacial free energy and then decreases beyond the critical size, indicating that clusters larger than this size are thermodynamically favored to grow spontaneously. A shift in the change of nucleation free energy is observed between the cluster sizes of three and five due to the relatively stable tetramer water structure. In Figure 3a, the nucleation stages of water were observed using our all-atom reactive force field simulation. For *T* = 298.15 K and a gas number density of ρ_*v*_ = 6 × 710^–7^ Å^3^, the figure shows that we obtain a critical cluster size of n* = 20.

**Figure 3:**
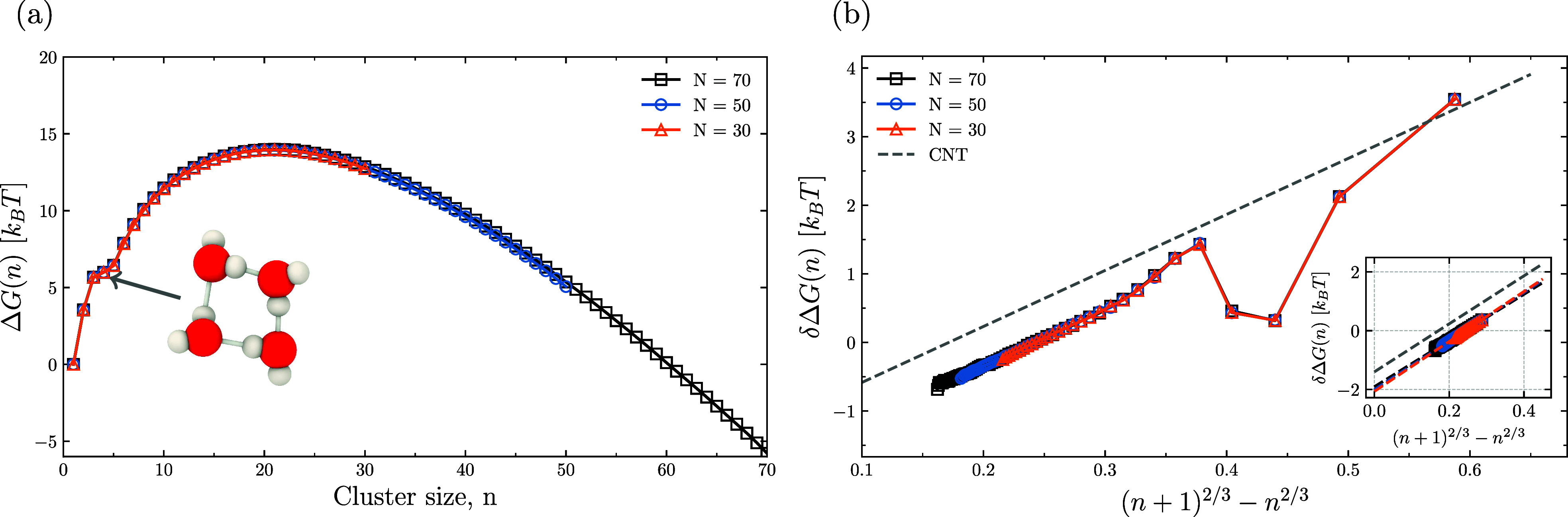
(a) Free energy of formation Δ*G* for water clusters as a function of cluster size *n* using our simple reactive water model at temperature *T* = 298.15 K using maximum cluster sizes of *N* = 30, 50, and 70. A shift in the free energy Δ*G* at cluster size *n* = 4 indicates the formation of a stable tetramer, consistent with observations in rigid water models. (b) Nucleation addition free energy, δΔ*G*, compared to predictions from classical nucleation theory (CNT). Inset: Linear fit of δΔ*G* for *n* > 9.

Figure 3b shows a plot of the addition free energy from the EB-AVBMC simulations using the Vashishta potential for three different maximum cluster sizes. We apply a linear (degree-one) fit to the classical nucleation theory (CNT) expression for δΔ*G*(*n*) given by,

$$\delta \Delta G\left(n\right)=\Delta \mu +\gamma {\left(\frac{36\pi }{\rho_{l}^{2}}\right)}^{1/3}\left({\left(n+1\right)}^{2/3}-n^{2/3}\right)$$ 20

The slope, *m*, of this linear fit can be used to evaluate both the quality of the saturated liquid density and surface tension, because *m* is given by

$$m=\gamma {\left(\frac{36\pi }{\rho_{l}^{2}}\right)}^{1/3}$$ 21

and thus allows for estimating the surface tension when the liquid density is known.

Similarly, the quality of the saturated gas phase density at equilibrium can be obtained from the intercept *b*:

$$b=\Delta \mu =-k_{B}T\ln\;\frac{\rho_{v}}{\rho_{\mathrm{eq}}}$$ 22

where *T* is temperature, ρ_v_ is the density of the ideal gas reservoir which is a user defined parameter, and ρ_eq_ is the gas density.

We obtained a reference slope of the linear fit by using experimental values of liquid density and surface tension, ρ_l_ = 0.997 g cm^–3^ and γ = 71.97 mN from NIST data,^44,45^ yielding a target slope of *m* = 8.166*k*_B_ *T*. A similar approach applies to gas density ρ_eq_. The experimental value of ρ_eq_ = 0.0255 kg m^–3^ ^45^ results in an intercept of *b* = −1.400*k*_B_ *T* at *T* = 298.15 K. Table 1 summarizes the linear regression slopes and intercepts from EB-AVBMC simulations, used to evaluate the liquid–vapor properties of our Vashishta parameter set for water.^42^

**Table 1:** Linear Regression Slope and Intercept of the EB-AVBMC Simulation Used to Determine the Quality of the Liquid–Vapor Properties of the Vashishta Parameter Set

cluster size (*N*)	*m* (*k*_B_ *T*)	*b* (*k*_B_ *T*)
30	8.48 ± 0.08	– 2.06 ± 0.02
50	8.26 ± 0.03	– 2.00 ± 0.01
70	7.88 ± 0.06	– 1.91 ± 0.01

The threshold cluster size at which linear behavior of the addition free energy is typically observed starts between 8 and 10.^43^ Thus, to ensure linearity, we applied a first-order polynomial fit only to cluster sizes greater than 9. Figure 3b suggests that using a maximum cluster size of 30 in the simulation yields a good estimate, with only minor deviations observed (as highlighted in the inset) compared to simulations with larger maximum cluster sizes of 50 and 70. Table 1 presents the intercepts and slopes obtained for various cluster sizes, which clearly show that both the slope (*m*) and intercept (*b*) depend on the maximum cluster size, *N*. As *N* increases, *m* decreases, and *b* increases. *N* = 30 seems like a good compromise since it yields slopes and intercepts close to the values for larger maximum cluster sizes, without requiring as long simulation times to adequately explore the configuration space. This trend underscores a critical consideration in cluster-based simulations: the choice of maximum cluster size represents a trade-off between computational feasibility and accuracy. Smaller *N* values allow for more extensive sampling of the configuration space within a given simulation time. However, they may introduce finite-size effects that lead to inaccuracies in the properties estimated from the simulation. This discrepancy is expected as CNT assumes that microscopic clusters possess the same constant surface tension as a macroscopic planar interface. A more refined description would incorporate a size-dependent surface tension, γ­(*r*), often described by the Tolman correction.^46^ In this case, the smaller *N* = 30 simulation slightly overestimates the slope *m*, which leads to an overestimated surface tension or an underestimated saturated liquid density. The intercept is overestimated, hence we expect an overestimated gas density. Conversely, larger *N* values are expected to be more representative of the bulk, but achieving adequate statistical sampling has become more challenging. A cluster size of about 30–50 molecules can provide a quick qualitative estimate of both surface tension and gas density, facilitating fast preliminary assessments before more extensive simulations.

To verify the accuracy of the EB-AMBMC calculations of our model’s saturated liquid density and gas density, as well as its surface tension, we performed a large molecular dynamics simulation of a liquid–vapor slab at 298.15 K. The resulting saturated liquid density was 1.0077(8) g cm^–3^, which is in good agreement with the reference value of 0.997 g cm^–3^. The simulated saturated gas density was 3(3) × 10^–5^ g cm^–3^, which is reasonably close to the experimental value of 2.559 × 10^–5^ g cm^–3^. We obtained a surface tension of γ = 56(7) mN m^–1^, which is consistent with rigid water models such as TIP4P (54.7 mN m^–1^) and SPC (53.4 mN m^–1^) at *T* = 298.15 K,^47^ but these are all lower than the experimental value of 72 mN m^–1^.^44^

Although we have benchmarked the EB-AVBMC method at 298.15 K, it should be robustly applicable to other regimes, as its energy-biasing scheme is designed to overcome the kinetic trapping at low temperatures, while its formulation naturally handles high supersaturation through umbrella sampling.^40^

We have implemented the extended EB-AVBMC method for the Vashishta reactive force field, but applying it to other force fields should be straightforward since only a calculation of the energy associated with performing trial moves is required. Polarizable or machine learning models may incur a higher computational cost. This is, however, likely to be acceptable, given that our Vashishta EB-AVBMC simulations were run on 1 CPU core each.

## Conclusions

In this work, we extended the EB-AVBMC algorithm for an all-atom force field by modifying the acceptance criteria and introducing constraints on the deletion move. With these modifications to the translation and swap moves, we computed nucleation free energies for our reactive water model and found them to be consistent with those of a rigid water model. Although this EB-AVBMC method has been specifically designed for Vashishta force field parametrization, a similar approach can be utilized for any all-atom force field. This modified EB-AVBMC method can also be applied to other existing central force fields.^48,49^ The ability to simulate nucleation phenomena using a reactive water model with the EB-AVBMC algorithm can be a useful tool to compute the liquid–vapor properties of other reactive water models and for studying the nucleation of liquid water under various thermodynamic conditions.

## Supplementary Material



## Notes

Water Structure, water model parameter set, and the hydrogen swapping algorithm (PDF)

## References

[1] Tang M., Cziczo D. J., Grassian V. H. (2016). Interactions of water with mineral
dust aerosol: water adsorption, hygroscopicity, cloud condensation,
and ice nucleation. Chem. Rev..

[2] Sleutel M., Lutsko J., Van Driessche A. E., Durán-Olivencia M. A., Maes D. (2014). Observing classical
nucleation theory at work by monitoring phase
transitions with molecular precision. Nat. Commun..

[3] Parks C., Koswara A., DeVilbiss F., Tung H.-H., Nere N. K., Bordawekar S., Nagy Z. K., Ramkrishna D. (2017). Solubility
curves and nucleation rates from molecular dynamics for polymorph
prediction–moving beyond lattice energy minimization. Phys. Chem. Chem. Phys..

[4] Harano K., Homma T., Niimi Y., Koshino M., Suenaga K., Leibler L., Nakamura E. (2012). Heterogeneous nucleation of organic
crystals mediated by single-molecule templates. Nature materials.

[5] Luo B., Wang Z., Curk T., Watson G., Liu C., Kim A., Ou Z., Luijten E., Chen Q. (2023). Unravelling crystal
growth of nanoparticles. Nature Nanotechnol..

[6] Visco D. P., Kofke D. A. (1998). Vapor–liquid
equilibria and
heat effects of hydrogen fluoride from molecular simulation. J. Chem. Phys..

[7] Yasuoka K., Matsumoto M. (1998). Molecular dynamics of homogeneous nucleation in the
vapor phase. I. Lennard-Jones fluid. J. Chem.
Phys..

[8] ten
Wolde P. R., Frenkel D. (1998). Computer simulation study of gas–liquid
nucleation in a Lennard-Jones system. J. Chem.
Phys..

[9] ten
Wolde P. R., Frenkel D. (1998). Numerical study of gas–liquid
nucleation in partially miscible binary mixtures. J. Chem. Phys..

[10] Kusaka I., Wang Z.-G., Seinfeld J. (1998). Direct evaluation of the equilibrium
distribution of physical clusters by a grand canonical Monte Carlo
simulation. J. Chem. Phys..

[11] Chen B., Siepmann J. I. (2000). A novel Monte Carlo
algorithm for simulating strongly
associating fluids: Applications to water, hydrogen fluoride, and
acetic acid. J. Phys. Chem. B.

[12] Chen B., Siepmann J. I., Oh K. J., Klein M. L. (2001). Aggregation-volume-bias
Monte Carlo simulations of vapor-liquid nucleation barriers for Lennard-Jonesium. J. Chem. Phys..

[13] Oh K., Zeng X. C. (2000). A small-system ensemble Monte Carlo simulation of supersaturated
vapor: Evaluation of barrier to nucleation. J. Chem. Phys..

[14] Chen B., Siepmann J. I., Oh K. J., Klein M. L. (2002). Simulating vapor–liquid
nucleation of n-alkanes. J. Chem. Phys..

[15] Merikanto J., Vehkamäki H., Zapadinsky E. (2004). Monte Carlo simulations of critical
cluster sizes and nucleation rates of water. J. Chem. Phys..

[16] Angélil R., Diemand J., Tanaka K. K., Tanaka H. (2014). Properties of liquid
clusters in large-scale molecular dynamics nucleation simulations. J. Chem. Phys..

[17] Angélil R., Diemand J., Tanaka K. K., Tanaka H. (2015). Homogeneous SPC/E water
nucleation in large molecular dynamics simulations. J. Chem. Phys..

[18] Allen, M. P. ; Tildesley, D. J. Computer simulation of liquids; Oxford University Press, 2017.

[19] Wierzchowski S., Kofke D. A. (2001). A general-purpose
biasing scheme for Monte Carlo simulation
of associating fluids. J. Chem. Phys..

[20] Busch N., Wertheim M., Chiew Y., Yarmush M. (1994). A Monte Carlo method
for simulating associating fluids. J. Chem.
Phys..

[21] Busch N., Wertheim M., Yarmush M. (1996). Monte Carlo
simulation of n-member
associating fluids: Application to antigen–antibody systems. J. Chem. Phys..

[22] Tsangaris D. M., de Pablo J. J. (1994). Bond-bias simulation of phase equilibria for strongly
associating fluids. J. Chem. Phys..

[23] Visco D. P., Kofke D. A. (1999). Modeling
the Monte Carlo simulation
of associating fluids. J. Chem. Phys..

[24] Chen B., Siepmann J. I. (2001). Improving the Efficiency
of the Aggregation- Volume-
Bias Monte Carlo Algorithm. J. Phys. Chem. B.

[25] Torrie G. M., Valleau J. P. (1974). Monte Carlo free
energy estimates using non-Boltzmann
sampling: Application to the sub-critical Lennard-Jones fluid. Chem. Phys. Lett..

[26] McKenzie M. E., Chen B. (2006). Unravelling the peculiar nucleation
mechanisms for non-ideal binary
mixtures with atomistic simulations. J. Phys.
Chem. B.

[27] Nellas R.
B., McKenzie M. E., Chen B. (2006). Probing the nucleation mechanism
for the binary n-nonane/1-alcohol series with atomistic simulations. J. Phys. Chem. B.

[28] Loeffler T. D., Sepehri A., Chen B. (2015). Improved monte carlo scheme for efficient
particle transfer in heterogeneous systems in the grand canonical
ensemble: Application to vapor–liquid nucleation. J. Chem. Theory Comput..

[29] Loeffler T. D., Chan H., Sasikumar K., Narayanan B., Cherukara M. J., Gray S., Sankaranarayanan S. K. (2019). Teaching
an old dog new tricks: Machine learning an improved TIP3P potential
model for liquid–vapor phase phenomena. J. Phys. Chem. C.

[30] Kumar R., Knight C., Wick C. D., Chen B. (2015). Bringing reactivity
to the aggregation-volume-bias Monte Carlo based simulation framework:
water nucleation induced by a reactive proton. J. Phys. Chem. B.

[31] Vashishta P., Kalia R. K., Rino J. P., Ebbsjö I. (1990). Interaction
potential for SiO2: A molecular-dynamics study of structural correlations. Phys. Rev. B.

[32] Vashishta P., Kalia R. K., Nakano A., Rino J. P. (2007). Interaction
potential
for silicon carbide: A molecular dynamics study of elastic constants
and vibrational density of states for crystalline and amorphous silicon
carbide. J. Appl. Phys..

[33] Branicio P.
S., Rino J. P., Gan C. K., Tsuzuki H. (2009). Interaction potential
for indium phosphide: a molecular dynamics and first-principles study
of the elastic constants, generalized stacking fault and surface energies. J. Phys.: Condens. Matter.

[34] Stillinger F. H. (1963). Rigorous
basis of the frenkel-band theory of association equilibrium. J. Chem. Phys..

[35] Torrie G. M., Valleau J. P. (1977). Nonphysical sampling distributions in Monte Carlo free-energy
estimation: Umbrella sampling. J. Comput. Phys..

[36] Kumar S., Rosenberg J. M., Bouzida D., Swendsen R. H., Kollman P. A. (1992). The weighted
histogram analysis method for free-energy calculations on biomolecules.
I. The method. Journal of computational chemistry.

[37] Siepmann J. I., Frenkel D. (1992). Configurational bias
Monte Carlo: a new sampling scheme
for flexible chains. Mol. Phys..

[38] Rosenbluth M. N., Rosenbluth A. W. (1955). Monte Carlo
calculation of the average extension of
molecular chains. J. Chem. Phys..

[39] Heijmans K., Tranca I. C., Chang M.-W., Vlugt T. J., Gaastra-Nedea S. V., Smeulders D. M. (2021). Reactive
Grand-Canonical Monte Carlo Simulations for
Modeling Hydration of MgCl2. ACS omega.

[40] Chen B., Siepmann J. I., Klein M. L. (2005). Simulating
Vapor- Liquid Nucleation
of Water: A Combined Histogram-Reweighting and Aggregation-Volume-Bias
Monte Carlo Investigation for Fixed-Charge and Polarizable Models. J. Phys. Chem. A.

[41] Loeffler, T. D. Advanced Monte Carlo Methods for the Study of Nucleation; Louisiana State University and Agricultural and Mechanical College, 2016.

[42] Camposano A. V. C., Nordhagen E. M., Sveinsson H. A., Malthe-So̷renssen A. (2025). Genetic Algorithm
Workflow for Parameterization of a Water Model Using the Vashishta
Force Field. J. Phys. Chem. B.

[43] Merikanto J., Zapadinsky E., Lauri A., Vehkamäki H. (2007). Origin of
the Failure of Classical Nucleation Theory: Incorrect Description
of the Smallest Clusters. Phys. Rev. Lett..

[44] Linstorm P. (1998). NIST chemistry
webbook, NIST standard reference database number 69. J. Phys. Chem. Ref. Data, Monograph.

[45] Lemmon, E. W. ; Bell, I. H. ; Huber, M. L. ; McLinden, M. O. Thermophysical Properties of Fluid Systems. NIST Chemistry WebBook, 2024; 10.18434/T4D303, Retrieved November 8, 2024.

[46] Tolman R. C. (1949). The effect
of droplet size on surface tension. J. Chem.
Phys..

[47] Chen F., Smith P. E. (2007). Simulated surface
tensions of common water models. J. Chem. Phys..

[48] Lemberg H. L., Stillinger F. H. (1975). Central-force model for liquid water. J. Chem. Phys..

[49] Bresme F. (2001). Equilibrium
and nonequilibrium molecular-dynamics simulations of the central force
model of water. J. Chem. Phys..

