# Supplementary material for: An Extended Energy-Biased Aggregation-Volume-Bias Monte Carlo (EB-AVBMC) Method for Nucleation Simulation of a Reactive Water Potential
Source: J Chem Theory Comput. 2025 Jul 4;21(14):6769–76. doi: 10.1021/acs.jctc.5c00722 (PMC12288005; doi:10.1021/acs.jctc.5c00722)
Supplement: Supplementary file 1 [file ct5c00722_si_001.pdf]

Supporting Information: An Extended Energy-Biased Aggregation-Volume-Bias Monte Carlo (EB-AVBMC) method for Nucleation Simulation of a Reactive Water Potential

Anthony Val C. Camposano,[†] Even Marius Nordhagen,[†,‡] Anders Malthe-Sørenssen,[¶] and Henrik Andersen Sveinsson[*,†]

†*The Njord Centre, Department of Physics, University of Oslo, Sem Sælands vei 24, NO-0316, Oslo, Norway*

‡*Norwegian Meteorological Institute, NO-0313, Oslo, Norway*

¶*The Njord Centre, Department of Physics, University of Oslo, Sem Sælands vei 24, NO-0313, Oslo, Norway*

E-mail: h.a.sveinsson@fys.uio.no

Water Structure

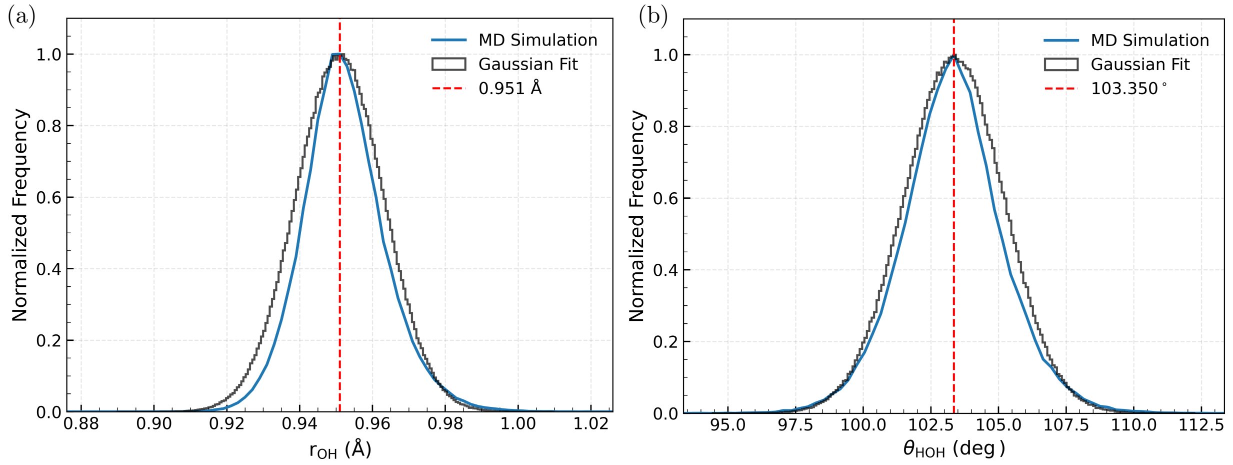

Figure S1: Comparison of an NVT molecular dynamics (MD) simulation data at a temperature of 298.15 K and Gaussian fits for (a) the O−H bond distance (r_{OH}) and (b) the H−O−H bond angle (θ_{HOH}) in water vapor. The structure of the inserted water molecule during the AVBMC simulation is sampled from these two Gaussian distribution. The MD simulation data (blue lines) are normalized to their maximum frequency, and the Gaussian fits (black lines) are generated using the Box-Muller algorithm,[S1] also normalized for comparison. Vertical red dashed lines indicate the peak positions at $0.951\mathring{A}$ for r_{OH} and 103.350° for θ_{HOH}, representing the most probable values from the simulations.

Water Model Parameter Set

Table S1: Two-body and three-body parameter set of the Vashishta water model.[S2]

Z_{O} (e)	Z_{H} (e)	r_{1s} (Å)	r_c (Å)
-0.943117	0.471558	4.477392	5.5

	H−H	H−O	O−O
η_{ij}	7.391415	13.237354	7.617388
r_{4s} (Å)	1.948928	1.661659	1.965379
H_{ij} (eV Å$^{\eta}$)	0.0	0.450836	1232.69421
D_{ij} (eV Å^4)	0.0	1.428122	91.090399
W_{ij} (eV Å^6)	0.0	0.229314	10.599231

	B_{ijk} (eV)	θ_0 (deg)	ξ (Å)	r_0 (Å)
H−O−H	259.861109	100.147683	0.503514	1.232741
O−H−O	0	0	0	0

Hydrogen Swapping Algorithm

Algorithm 1 Hydrogen Atom Swapping: This algorithm processes each water molecule, reassigning hydrogen atoms based on their proximity, ensuring that each oxygen atom is assigned at most two of the nearest hydrogen.

```
procedure HydrogenSwap(MolArray, NPART)
    for iMol = 1 to NPART do
        iIndx ← MolArray%mol(iMol)%indx
        (O_1x, O_1y, O_1z) ← Oxygen coordinates of reference molecule
        Compute O_1−H distances r1_ij and r2_ij (hydrogen 1 and 2)
        for update = 1 to 2 do
            shortest_dist ← ∞
            shortest_indx ← iIndx
            for jMol = 1 to NPART do
                if jMol ≠ iIndx then
                    (O_2x, O_2y, O_2z) ← Oxygen coordinates of the compared molecule
                    for jAtom = 2 to 3 do
                        Compute O_1−H_jAtom distance r_ij1 and O_2−H_jAtom distance r_ij2
                        if r_ij1 < r_ij2 and r_ij1 ≤ shortest_dist then
                            shortest_indx ← jMol
                            shortest_dist ← r_ij1
                            shortest_jAtom ← jAtom
                        end if
                    end for
                end if
            end for
            if shortest_dist ≠ ∞ then
                if shortest_dist ≤ r1_ij and shortest_dist ≥ r2_ij then
                    Swap hydrogen 1 with shortest_jAtom hydrogen
                else if shortest_dist ≥ r1_ij and shortest_dist ≤ r2_ij then
                    Swap hydrogen 2 with shortest_jAtom hydrogen
                else if shortest_dist ≤ r1_ij and shortest_dist ≤ r2_ij then
                    if r1_ij ≤ r2_ij then
                        Swap hydrogen 2 with shortest_jAtom hydrogen
                    else
                        Swap hydrogen 1 with shortest_jAtom hydrogen
                    end if
                end if
            end if
        end for
    end for
end procedure
```

References

(S1) Box, G. E.; Muller, M. E. A note on the generation of random normal deviates. *The annals of mathematical statistics* **1958**, *29*, 610–611.

(S2) Camposano, A. V. C.; Nordhagen, E. M.; Sveinsson, H. A.; Malthe-Sørenssen, A. Genetic Algorithm Workflow for Parameterization of a Water Model Using the Vashishta Force Field. *The Journal of Physical Chemistry. B* **2025**, *129*, 1331.
